# Acute Renal Failure Caused by Undiagnosed Pelvic Organ Prolapse in a Postmenopausal Woman: A Diagnosis Not to Be Missed

**DOI:** 10.7759/cureus.44513

**Published:** 2023-09-01

**Authors:** Mansour Khaleel, Mohammed Ayyad, Maram Albandak, Nabil C. N. Khalil, Sari M. A. Abu Taleb

**Affiliations:** 1 Internal Medicine, Al-Makassed Charitable Society Hospital, Jerusalem, PSE; 2 Internal Medicine, Al-Quds University, Jerusalem, PSE; 3 Internal Medicine, I. K. Akhunbaev Kyrgyz State Medical Academy, Bishkek, KGZ

**Keywords:** aki, obstructive uropathy, pelvic organ prolapse, obstructive renal failure, hydronephrosis, postmenopausal woman, azotemia, anuria, obstructive pyelonephritis

## Abstract

Pelvic organ prolapse (POP) is a common condition mainly affecting postmenopausal women, characterized by the descent of pelvic organs through the vaginal canal. While often asymptomatic, POP can manifest with various symptoms such as a painless bulge or pressure sensation, abdominal pain, urinary complaints, and discomfort during intercourse. Severe cases can lead to urinary tract obstruction, hydronephrosis, and renal dysfunction. This case study presents an elderly female with bilateral severe hydronephrosis and pyelonephritis due to undiagnosed POP. Imaging revealed obstructive uropathy resulting from bilateral ureteric compression caused by cystocele and uterine prolapse. The patient's condition improved with antibiotics and supportive management. A vaginal hysterectomy was performed, which led to the resolution of the urinary tract obstruction. This case emphasizes the importance of considering POP in elderly women with urinary symptoms and the need for proactive screening. It highlights the significance of appropriate management to prevent irreversible renal damage. Different treatment modalities, including surgery and pessaries, are discussed, to emphasize the significance of tailoring treatments to individual patient characteristics.

## Introduction

Pelvic organ prolapse (POP) is characterized by the downward displacement of pelvic organs, causing them to protrude through the vaginal canal. This condition is prevalent among elderly women and is caused by a complex pathophysiology related to anatomical, hormonal, and age-related disturbances [1]. While often asymptomatic, individuals may experience manifestations such as a sensation of vaginal fullness, discomfort in the lower back or abdomen, dysuria, dyspareunia, increased urinary urgency and frequency, and difficulty in urination [2]. Additionally, POP can cause obstruction in the urinary tract, leading to diminished urine flow rates and an elevation in residual post-void urine volumes [3]. Prompt diagnosis and management of POP-induced obstruction is vital to prevent the development of acute kidney injury (AKI) or chronic renal failure that could potentially progress to end-stage renal disease [4]. Despite the recognized association between POP and hydronephrosis, instances of severe hydronephrosis and impaired kidney function remain infrequent [5]. This report outlines the case of a 77-year-old female patient who presented with flank pain. Subsequent evaluation revealed bilateral severe hydronephrosis and pyelonephritis related to undiagnosed pelvic organ prolapse.

## Case presentation

A 77-year-old multiparous female presented to the hospital complaining of bilateral flank pain and fever of five days duration. The pain manifested as intermittent episodes, recurring several times over the past days and lasting for up to 15 minutes during each occurrence. The pain exhibited colicky qualities and radiated towards the groin area. The patient subjectively rated the severity of the pain as six out of 10. In addition to the pain, the patient reported sensations of burning during urination, along with nausea, vomiting, rigors, and chills. She revealed that she had experienced decreased urination in the last two days. Notably, she had been experiencing pelvic heaviness and increased urinary frequency for several years. Her medical records indicated multiple hospital admissions due to recurring urinary tract infections. She subsequently underwent stone protocol computed tomography (CT) imaging, urinalysis, and urine microscopy, all of which were unremarkable for stones. She also denied any urine discoloration or previous symptoms consistent with kidney stones during her previous admissions. Her medical history was significant for hypertension, diabetes mellitus, and chronic kidney disease related to diabetes, which was characterized by a baseline creatinine level of 1.5 mg/dL. Obstetric history revealed a record of five uncomplicated vaginal deliveries and no history of abortion.

On physical examination, the patient was in obvious distress due to pain. She was conscious, oriented, and alert to time, place, person, and situation. Vital signs revealed a heart rate of 110 beats per minute (bpm), a blood pressure of 120/73 mm Hg, an oxygen saturation of 99% on room air, and a temperature of 38.9 degrees Celsius. Her abdomen was soft, not tender, and non-distended. However, there was significant suprapubic and costovertebral angle tenderness bilaterally. Pelvic examination revealed an apical and anterior compartment prolapse of the uterus (grade IV), and a grade III cystocele without obvious urinary leakage. The urethral meatus was easily visualized. The remainder of the examination did not reveal any notable abnormalities.

Laboratory investigations showed a white count of 11.7 × 10^9^/L, a low hemoglobin of 10.3 g/dL with a mean corpuscular volume (MCV) of 85.8, an elevated creatinine of 2 mg/dL, an erythrocyte sedimentation rate (ESR) of 65 mm/hr, and a blood urea nitrogen (BUN) of 35 mg/dL. Urinalysis revealed bacteriuria, pyuria (25-30 WBC/hpf), nitrate positivity, and microscopic hematuria (20-25 RBC/hpf), with a urine specific gravity of 1.01. Other laboratory investigations were normal. Urine culture was positive for E. Coli. Renal and bladder ultrasound revealed severe bilateral hydronephrosis with thinning of the renal parenchyma. Additionally, a diminished distinction between the corticomedullary regions was observed. The measurements obtained were as follows: the right renal pelvis had a diameter of 4.3 cm, the upper segment of the right ureter measured 2.5 cm, the diameter of the left renal pelvis was 4 cm, and the proximal portion of the left ureter displayed a diameter of 2.3 cm. Subsequently, a Foley’s catheter was inserted, and the patient was started on ceftriaxone and aggressive fluid therapy.

**Figure 1:**
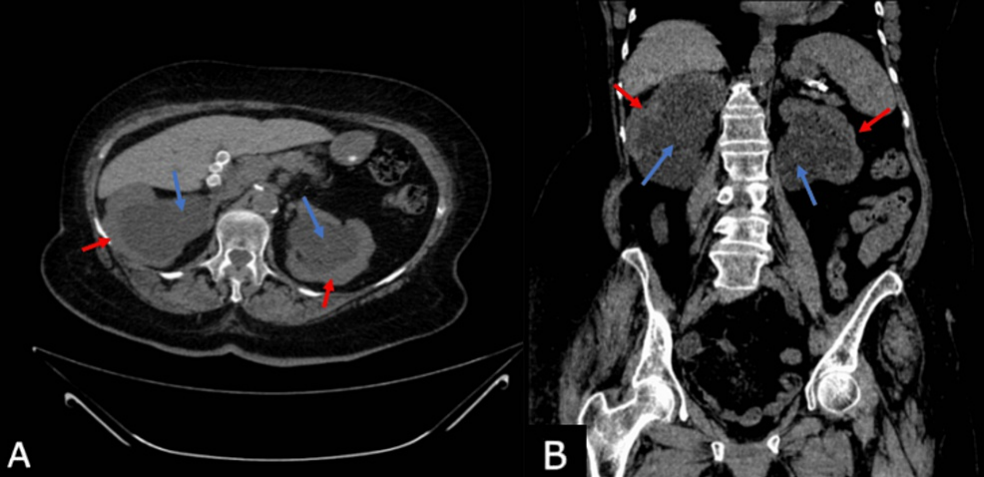
CT scans of the abdomen; coronal and axial views. Axial and coronal CT scans of the abdomen without IV contrast (A and B) demonstrating severe bilateral hydronephrosis (blue arrows) with thinning of the renal cortex bilaterally (red arrows).

**Figure 2:**
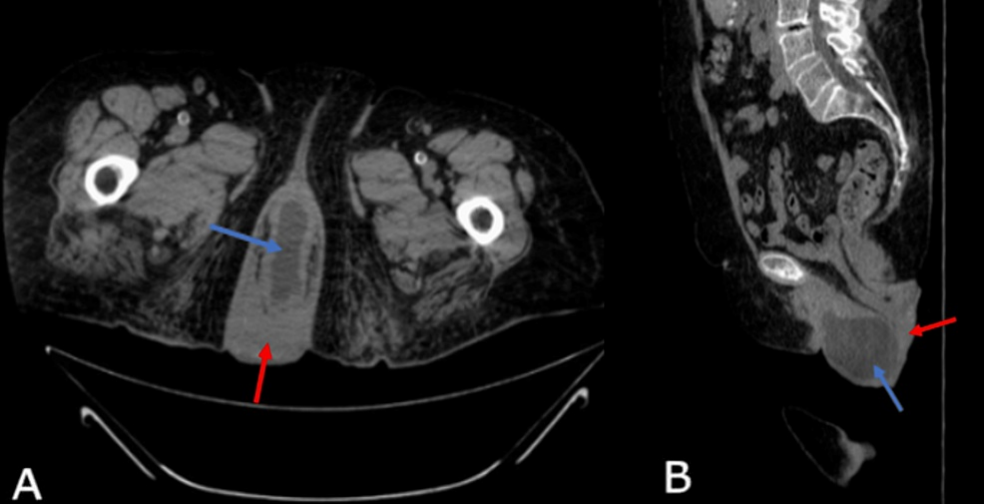
CT scans of the abdomen without contrast; axial and sagittal views. Axial and sagittal abdominal CT scans without IV contrast (A and B) demonstrating an abnormally positioned urinary bladder below the pubic symphysis (blue arrows indicative of a grade III cystocele), as well as prolapse of the uterus (red arrows).

**Figure 3:**
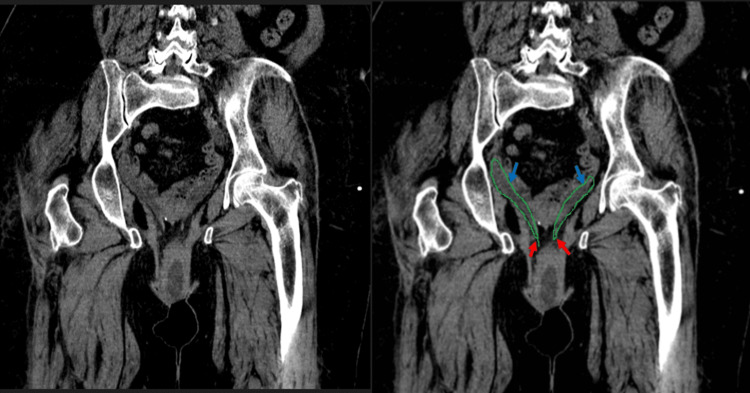
Coronal CT scan of the abdomen showing hydronephrosis caused by external compression. Original and outlined coronal abdominal CT scan without intravenous contrast displaying both ureters (outlined by the green line), with notable dilation (indicated by blue arrows) along with tapering constriction of the distal bilateral segments (highlighted by red arrows), suggestive of external compression.

Furthermore, the abdomen and pelvis CT scan showed severe bilateral hydronephrosis with marked tortuosity and thinning of the renal cortices (Figure 1). The ureters were markedly dilated along with a tapering constriction of the distal bilateral ureters, indicative of external compression (Figure 2). The CT scan also showed prolapse of the uterus and abnormal positioning of the urinary bladder below the pubic symphysis consistent with grade III cystocele (Figure 3). Both kidneys were in normal anatomical positions and no renal or ureteric stones were observed.

Based on these findings, the cystocele and uterine prolapse were identified as the primary contributors to the obstruction within the urinary tract, resulting in the development of urinary tract infection (UTI) and AKI. The presence of chronic bilateral compression at the ureteric orifices led to obstructive uropathy, causing bilateral hydronephrosis. Furthermore, the concurrent pyogenic infection likely exacerbated the hydronephrosis and contributed to the deterioration of the renal parenchyma, thereby amplifying the AKI. The patient's treatment regimen included antibiotics, intravenous fluids, and the maintenance of a catheter, which collectively led to gradual clinical improvement. After three days, the patient's condition stabilized, with gradual normalization of vital signs and a reduction in the hydronephrosis as evident on ultrasound. Notably, the patient's creatinine levels improved to 1.39 mg/dL, and her BUN decreased to 21.5 mg/dL. Following consultation with the gynecologic service, a decision was made to proceed with a vaginal hysterectomy coupled with anterior and posterior repair procedures to alleviate the obstruction. The patient retained a Foley's catheter and ultimately underwent the surgical intervention. Post-surgery, the patient displayed clinical improvement, and imaging studies confirmed the resolution of the urinary tract obstruction. Subsequently, the patient was discharged home without encountering any further complications.

## Discussion

Pelvic organ prolapse is a common condition that is infrequently associated with significant morbidity and mortality. Interestingly, roughly 3-6% of women are expected to eventually describe symptoms linked to POP, whereas approximately 50% of women receive a POP diagnosis through pelvic examination [6].

POP presentation may be an incidental diagnosis on physical exam, however, it can also present with lower abdominal or back pain, vaginal fullness, urinary symptoms, or painful intercourse [2]. Moreover, POP might pose a risk for renal complications as it can result in significant urinary tract obstruction, leading to a reduction in urinary flow rates and an increase in post-void residual volume [3]. Therefore, failure to diagnose or properly treat the condition can culminate in life-threatening complications, such as acute kidney injury that can progress to chronic renal failure with a possibility of developing end-stage renal disease [4].

While it is not uncommon for POP to result in urinary tract obstruction and hydronephrosis, instances of severe hydronephrosis and renal dysfunction could rarely occur [5]. In fact, the association between POP and hydronephrosis underscores the necessity for proactive screening in patients displaying hesitancy in reporting symptoms. Furthermore, a review article conducted by Siddique et al. highlighted that hydronephrosis exists in about 3.6% to 30.6% of POP patients, which is contingent upon the specific studies [7]. The review also demonstrated that uterovaginal prolapse contributed more significantly to hydronephrosis, particularly in severe cases, than vaginal vault prolapse. Nonetheless, none of the studies managed to reveal a substantial link between the stage of hydronephrosis and the severity or duration of the prolapse [7].

In our case, the patient was an elderly female presenting with pronounced cystocele and uterine prolapse, resulting in urinary outlet obstruction that subsequently precipitated her acute kidney injury. Notably, our patient's history of recurring urinary tract infections and her hesitance to disclose complaints of urinary dysfunction to her physician raises the concern that her POP diagnosis has been overlooked for a long time. This highlights the importance of conducting a thorough physical examination, even in the absence of a comprehensive medical history due to the possibility of patients' embarrassment to report their symptoms, as was apparent in our case. We posit that our patient's uropathy stemmed from a ureteral obstruction, substantiated by imaging evidence indicating bilateral hydronephrosis with thinning of the renal parenchyma, coupled with markedly dilated and tortuous ureters. Therefore, a gynecologic exam is crucial for females exhibiting symptoms of urinary outlet obstruction or evidence of hydroureteronephrosis of uncertain origin, particularly in elderly patients, given the proportional rise in POP incidence with advancing age [4].

The treatment of POP-induced hydronephrosis should prioritize preventing complications involving the upper urinary tract, while simultaneously managing the patient’s symptoms [8]. Our patient became afebrile after the administration of antibiotics along with supportive treatment. Subsequently, a vaginal hysterectomy was performed, along with anterior and posterior repairs. Notably, the choice of surgical reduction may encompass either a suprapubic or vaginal approach, with a preference for incorporating hysterectomy whenever feasible [8]. The choice for a vaginal versus abdominal hysterectomy should be individualized on a case-by-case basis, based on the severity of the prolapse and characteristics of the patient. Given the severity of our patient’s complaints and longstanding symptoms, vaginal approach with hysterectomy was deemed appropriate. Research conducted at Rush-Presbyterian-St. Luke's Medical Center demonstrated an 89% success rate in restoring urinary post-void residual volume following surgical repair of POP [3]. Conversely, surgery can be contraindicated for many reasons, however, if the patient does not desire reconstructive options, colpocleisis can be performed in elderly patients with multiple comorbidities as it can be performed under local anesthesia with a lower frequency of complications [9].

Nonetheless, some patients opt for conservative management because of personal preferences, limited treatment options, or contraindications to surgery. In cases where surgery is unsuitable, the insertion of a pessary presents a conservative alternative that merits consideration, especially in poor surgical candidates. Though uncommon, complications such as a foul-smelling vaginal discharge, fistulas, and erosions could develop [10]. Moslemi et al. documented two cases of women in their 80s with severe POP resulting in hydronephrosis and obstructive nephropathy. The first patient initially underwent the placement of percutaneous nephrostomy tubes bilaterally, and later a vaginal pessary was inserted. Meanwhile, the second patient underwent surgical intervention with temporary placement of double J stents. Both cases witnessed the resolution of hydronephrosis and normalization of creatinine levels [4]. A comparable conclusion was also reached by Young et al. who reported a patient in septic shock as a result of acute obstructive pyelonephritis attributable to POP [11]. The utilization of a ring pessary, coupled with the reduction of the prolapse in this patient, proved sufficient for the drainage of infected urine [12]. Furthermore, studies have also shown that manually reducing the prolapse is sufficient to provide outlet obstruction relief, leading to effective drainage of urine [13].

Conversely, Bae et al. reported the case of an elderly female complaining of anorexia and fatigue. Subsequent investigations revealed hydronephrosis caused by severe grade V uterine prolapse and an elevated creatinine measuring 12.35 mg/dl. Despite intensive management, the renal function progressively worsened and the patient developed end-stage renal disease [13]. Our case emphasizes the importance of maintaining a heightened suspicion for pelvic organ prolapse as the underlying cause of urinary obstruction in symptomatic patients exhibiting renal dysfunction. Timely correction of this anatomical obstruction could be imperative in some cases, as failure to do so promptly could lead to irreversible deterioration of renal function.

## Conclusions

In summary, pelvic organ prolapse can lead to kidney injury by causing blockage in the urethra or ureters. Our case underscores the need to maintain a vigilant consideration for POP when patients exhibit urinary problems. It also highlights the significance of thorough physical examinations, even when a comprehensive medical history is unavailable, as failing to promptly address the obstruction could result in permanent renal damage. On the other hand, the definitive treatment of POP usually includes surgery, however, the use of pessaries to rectify the organ prolapse is plausible in patients with contraindications or those who prefer conservative management. While both methods are associated with positive outcomes, an individualized approach taking into account patient characteristics, comorbidities, and personal preference should be utilized when deciding between the two options. Additional research is needed to conduct a comparative analysis of the efficacy and safety of these treatment modalities.
